# Depsipeptide Analogues of Gly-Ala-Gly: Proton Localization and Effects on Collision-Induced Dissociation

**DOI:** 10.1021/jasms.5c00371

**Published:** 2025-12-22

**Authors:** Brison A. Shira, Elin C. Herndon, Julianna E. DeMauro, Michael W. Giuliano, Jay G. Forsythe

**Affiliations:** Department of Chemistry and Biochemistry, 2343 College of Charleston, Charleston, South Carolina 29424, United States

**Keywords:** depsipeptide, peptide, MS/MS, CID, mobile proton

## Abstract

Depsipeptides are peptides that contain both amino acid and hydroxy acid residues. In this study, we sought to investigate how terminal hydroxyl groups and/or backbone ester linkages from hydroxy acid residues in depsipeptides affected collision-induced dissociation (CID). The canonical tripeptide glycine-alanine-glycine (GAG) was compared to all three of its depsipeptide analogues: glycolic acid-AG (gAG), G-lactic acid-G (GaG), and GA-glycolic acid (GAg). Experimental data was supported by density functional theory (DFT) calculations to gain insight into which sites on the molecules have sufficient proton affinity (PA) to localize the proton and the resulting charge-directed fragmentation processes.

## Graphical abstract

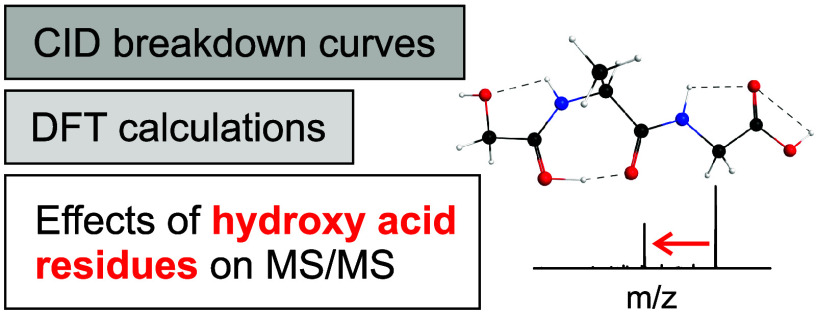

## Introduction

Tandem mass spectrometry (MS/MS) is a standard technique for peptide detection and sequencing.^1^ Collision-induced dissociation (CID), also known as collision-activated dissociation (CAD), generates predictable fragmentations giving sequence ions when directed at proteinogenic peptides.^2^ For example, common b- and y-type fragments correspond to cleavages of amide/peptide bonds between amino acid residues. CID fragmentation pathways are often highly reproducible across instrument types and models.

CID is often understood in the context of mobile proton theory, wherein for a peptide [M+nH]^n+^ ion, there exist multiple Brønsted-basic sites that might localize charge-carrying protons.^3^ Protonation sites on the N-terminal amine, basic side chains, and/or backbone carbonyls drive the formation of charge-directed fragment ions; that is, proton localization lowers the energy of the lowest unoccupied molecular orbital (LUMO), priming that site for intramolecular nucleophilic attack, causing fragmentation.^4–8^

Our motivation for examining CID fragmentation of peptide-like oligomers extends from our interest in prebiotic chemistry and astrobiology. Various proposals exist to explain the emergence of peptides and proteins that comprise modern biology.^9,10^ Past work has explored evaporative wet–dry cycling as a source of abiotic/prebiotic depsipeptides—copolymers of amino acids and hydroxy acids—that increase in peptide character over time.^11^ Thermodynamically favorable O-to-N acyl transfer drives this process at low water activity; intermediate ester linkages are activated for nucleophilic attack and peptide bond formation.

As we analyzed depsipeptide products of our prebiotic chemistry experiments by MS/MS, we attempted to apply conventions of traditional CID peptide sequencing and observed some differences in fragmentation—more pronounced in positive-ion mode than negative-ion mode (linear depsipeptides and peptides have identical C-termini, and thus similar deprotonation).^12,13^ It is notable that CID-based MS/MS strategies have been used to detect peptides and peptide-like products in other model prebiotic contexts as well, such as microdroplets.^14–16^

To augment our understanding of positive-ion mode depsipeptide CID with a long-term goal of incorporating such information into automated, high-accuracy sequencing algorithms akin to those in proteomics, we undertook a detailed study of the depsipeptide analogues of Gly-Ala-Gly tripeptide (GAG): glycolic acid-Ala-Gly (gAG), Gly-lactic acid-Gly (GaG), and Gly-Ala-glycolic acid (GAg) (Scheme 1). According to the nitrogen rule, GAG has an odd neutral mass of 203 Da ([M + H]^+^ = 204 Da), and its depsipeptide analogues have even neutral masses of 204 Da ([M + H]^+^ = 205 Da).

**Scheme 1:**
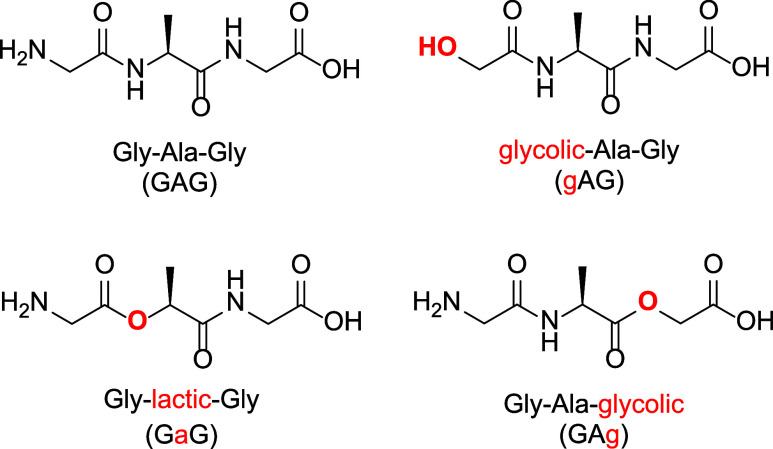
GAG and Its Depsipeptide Analogues: Atomic Substitutions Differentiating the Depsipeptides from the Peptide Are Colored Red

Hydroxy acid residues in depsipeptides are thought to complicate positive-mode CID fragmentation in several ways. First, “N”-terminal hydroxy acids (such as gAG) lack a primary amine, decreasing analyte gas-phase basicity and proton affinity (PA, which is the opposite of the enthalpy of the protonation reaction).^17–19^ Second, the smaller PA of depsipeptides’ ester carbonyl oxygens compared to peptides’ amide carbonyl oxygens, which gain electron density via a conjugated nitrogen lone pair, may influence proton mobility and localization across the backbone. Third, ester linkages lack the planarity of traditional amide/peptide linkages; this may influence proton localization or gas-phase conformational rigidity as well.

In this study, CID breakdown curves were generated for the [M + H]^+^ ion of a GAG control peptide (previously studied by Mookherjee and Armentrout^20,21^ ) and its three depsipeptide analogues. Experimental data were supported by density functional theory (DFT) computations. In lieu of rigorous Rice–Ramsperger–Kassel–Marcus (RRKM) theory (which requires calculations of transition state vibrations), we used a simpler, static computational chemistry approach designed to predict PA throughout each analyte [M + H]^+^ ion in full consideration of inductive effects and intramolecular hydrogen bonding.^18,22,23^ We found that, relative to the same sites in the GAG control peptide, PA was lowered in and adjacent to hydroxy acid residues, and this can often rationalize the CID fragments observed. By applying principles of mobile proton theory to depsipeptides, we gained insight into how proton localization and migration guide the interpretation of MS/MS data toward our goal to sequence depsipeptides of diverse monomer content.

## Methods

### Experimental Section

GAG (1 g; >99% purity), GaG (7 mg; 94.1% purity), and GAg (9 mg, TFA salt; 98.6% purity) were synthesized by BACHEM Americas (Torrence, CA) and used without further purification. The depsipeptide analogue gAG was synthesized in-house using a solution-phase synthesis approach (20 mg; ∼95% purity). Synthetic and characterization details are provided in SI Section I.

GAG, GaG, and GAg were constituted at 100 μM in a 50:50 water/methanol mixture with 0.1% formic acid. Because it lacks a free amine for protonation and has a lower ionization efficiency, gAG was constituted at 25.0 mM in 50:50 water to methanol with 0.1% formic acid. These solutions were subjected to electrospray ionization (ESI) on a Thermo LTQ Velos Pro linear quadrupole ion trap (LQIT) mass spectrometer. Positive-ion mode MS/MS experiments were conducted at various lab-frame collision energies, and these results are the principal basis of this study. The activation *q* was 0.250, and the activation time was 30.0 ms; He was the collision gas. See SI Section II for instrument parameters and SI Section III for full scan positive and negative mode mass spectra.

### Computational Methods

DFT calculations were used to simulate the analytes in Scheme 1 as [M + H]^+^ ions (calculations were performed using WebMO with a Gaussian 16 engine and B3LYP theory with basis set 6-311+G­(2d,p); additional details in SI Section IV).^24–28^ To investigate the role(s) of the mobile proton, we modeled the analytes associated with a proton at each of its polar heteroatoms and calculated their energy after geometry minimization. Replicating past approaches from the literature,^6,7,20,21,29–31^ variant structures were generated by forming various hydrogen-bonding networks (where two heteroatoms are within 3 Å of a hydrogen) across the analyte backbone.^32,33^ In other words, a suite of geometries was generated in which (i) a proton was added to each of the heteroatoms, (ii) each of the protonated sites was stabilized with different networks of intramolecular hydrogen bonds, and (iii) the set of all of these minimized structures was considered together.

This allowed us to build a potential energy surface (PES) where the energies of the proton localized at different backbone moieties could be compared across each of the analogues. The goal of these computations was to judge local PA in consideration of the inductive effects and intramolecular bonding. This information was used to rationalize the role of the charge direction in the observed fragmentation.

We did not use literature values of PAs for the functional groups comprising the peptide and depsipeptides because inductive effects and intramolecular hydrogen bonding are hypothesized to play a significant role in determining the actual PA of each location in the [M + H]^+^ ion.^16^ We used the PES to qualitatively interpret how hydroxy acid residues perturb the PA of the depsipeptide backbone relative to that of the peptide control, and influence fragment ion generation in CID breakdown curves.^34^

This approach allowed us to probe the conformational space of each compound without the need for calculations of transition state vibrations, which are required for more rigorous RRKM theory. Though this sacrifices quantitative thermodynamic insight, it served our goal to qualitatively understand how hydroxy acid residues in depsipeptides perturb PA, and thus fragmentation on the backbone.

## Results and Discussion

CID MS/MS spectra for GAG and its three depsipeptide analogues at 20 collision energy (lab-frame) are presented in Figure 1. Consistent with Armentrout,^20,21^ the dominant fragment of the GAG control peptide was b_2_.

**Figure 1:**
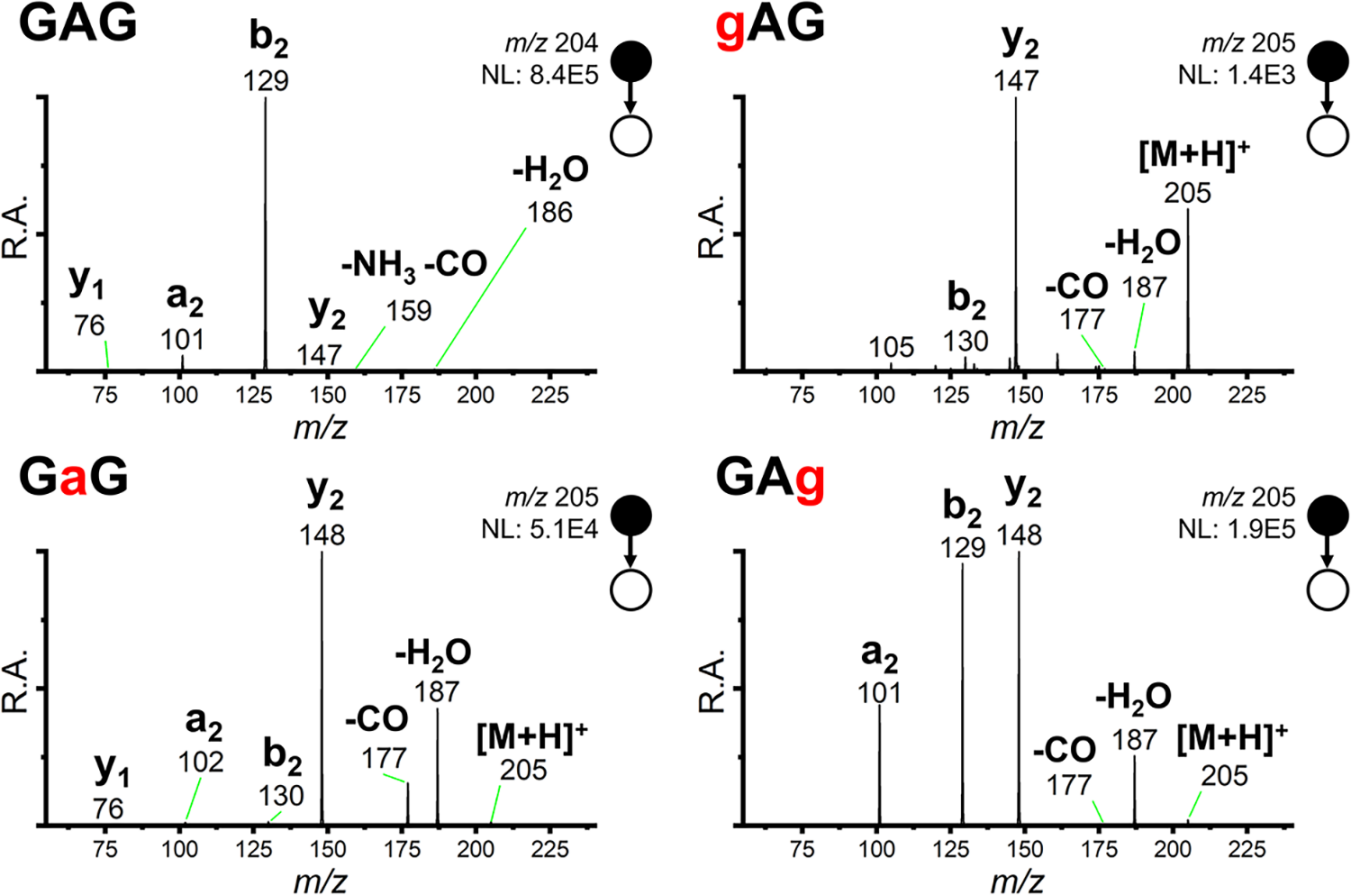
Tandem mass spectra for all four analytes of interest. All spectra were recorded using 20 collision energy (lab-frame/arb. units). R.A. is relative abundance, scaled to the most intense peak; NL is the normalization level, which indicates the arbitrary intensity units assigned to the most intense fragment ion.

Notable differences in the MS/MS spectra were observed for the three depsipeptide analogues. The gAG depsipeptide lacks a primary amine, strongly affecting its ionization efficiency; despite being sprayed from a solution 250 times more concentrated, its [M + H]^+^ ion and resulting fragments appeared at roughly 2 orders of magnitude lower intensity than the others. Both GaG and gAG generated prominent y_2_ fragment ions instead of b_2_ fragment ions. GAg generated prominent b_2_, y_2_, and a_2_ fragments.

It is worth restating that the only difference between each depsipeptide analogue and the GAG control was the replacement of one −NH for one −O (and a corresponding nominal mass difference of +1 Da). Moving the hydroxy acid residue across the backbone, thus, the −NH for −O replacement, induced major differences in CID fragmentation.

Breakdown curves from 0–40 collision energy (lab-frame) are shown in Figure 2. GAG control spectra (Figure 2a) were dominated by the b_2_ fragment, with minor contribution from the a_2_ fragment, which may be a subsequent fragmentation of b_2_.^7,35^ The gAG spectra (Figure 2b) had dominant y_2_ fragment ions, GaG spectra (Figure 2c) also had dominant y_2_ fragment ions with more water loss, and GAg spectra (Figure 2d) showed significant y_2_, b_2_, a_2_, and water-loss fragments.

**Figure 2:**
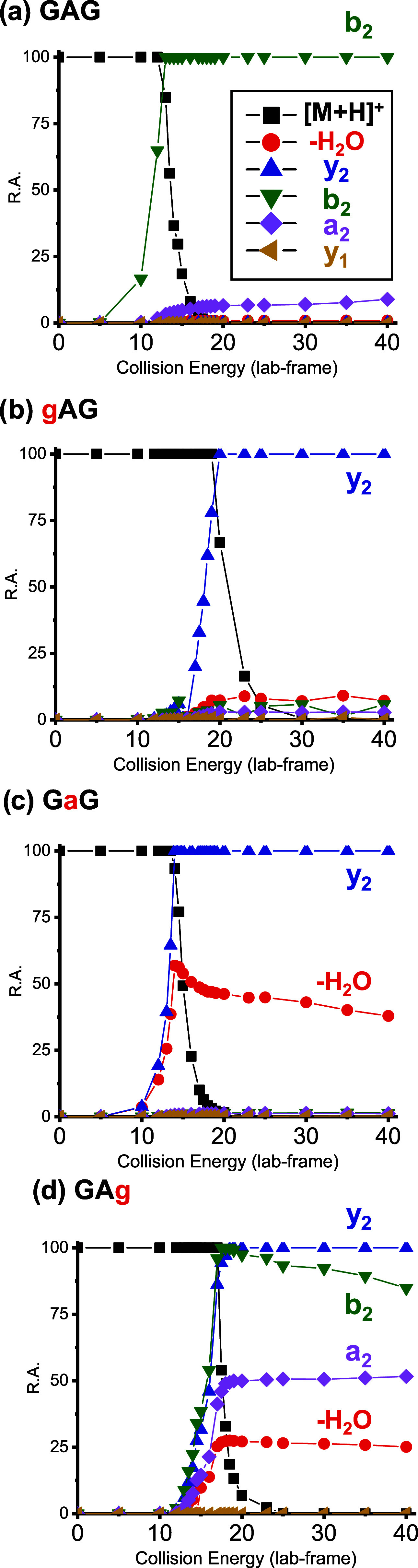
Breakdown curves for (a) GAG, (b) gAG, (c) GaG, and (d) GAg (0–40 collision energy, lab-frame). Major sequence ions and neutral-loss fragments are shown. GAG generated a dominant b_2_ fragment ion, whereas gAG generated a dominant y_2_ fragment ion. GaG generated strong y_2_ and water-loss fragments, and GAg generated a mixture of y_2_, b_2_, a_2_, and water-loss fragments.

The four analytes showed varying gas-phase stabilities. Collision energies at 50% parent ion R.A. (±95% CI), listed from highest to lowest, were: gAG, 20.9 (±0.2); GAg, 17.14 (±0.05); GaG, 15.10 (±0.66); and GAG, 13.77 (±0.06). Curves were fit using a Boltzmann distribution (all *R* ^2^ ≥ 0.992) and are provided in SI Section V.

Computation was used to support the breakdown curve data. Analyte ions were considered to be dynamic structures that could instantaneously localize the charge-carrying proton. PA throughout the [M + H]^+^ ion depends on networks of intramolecular hydrogen bonding that stabilize the charge; we refer to each of the points across the PES as a proton localization geometry (PLG).^36,37^ Low-energy PLGs are shown in Figure 3. Sites within 20 kJ/mol of energy minima (gray band) were considered most accessible. Slow heating in the LQIT mass analyzer made it likely that all sites within the gray band were accessible before fragmentation.^14,38–40^ Literature suggests that trapped ions during mass analysis can be approximated to ca. 300 K;^41^ at this temperature, thermodynamic barriers greater than 20 kJ/mol are relatively high (for *T* = 300 K and Δ*E* = 20 kJ/mol, the equilibrium constant K approaches 10^–4^ by the Gibbs/Boltzmann distribution).

**Figure 3:**
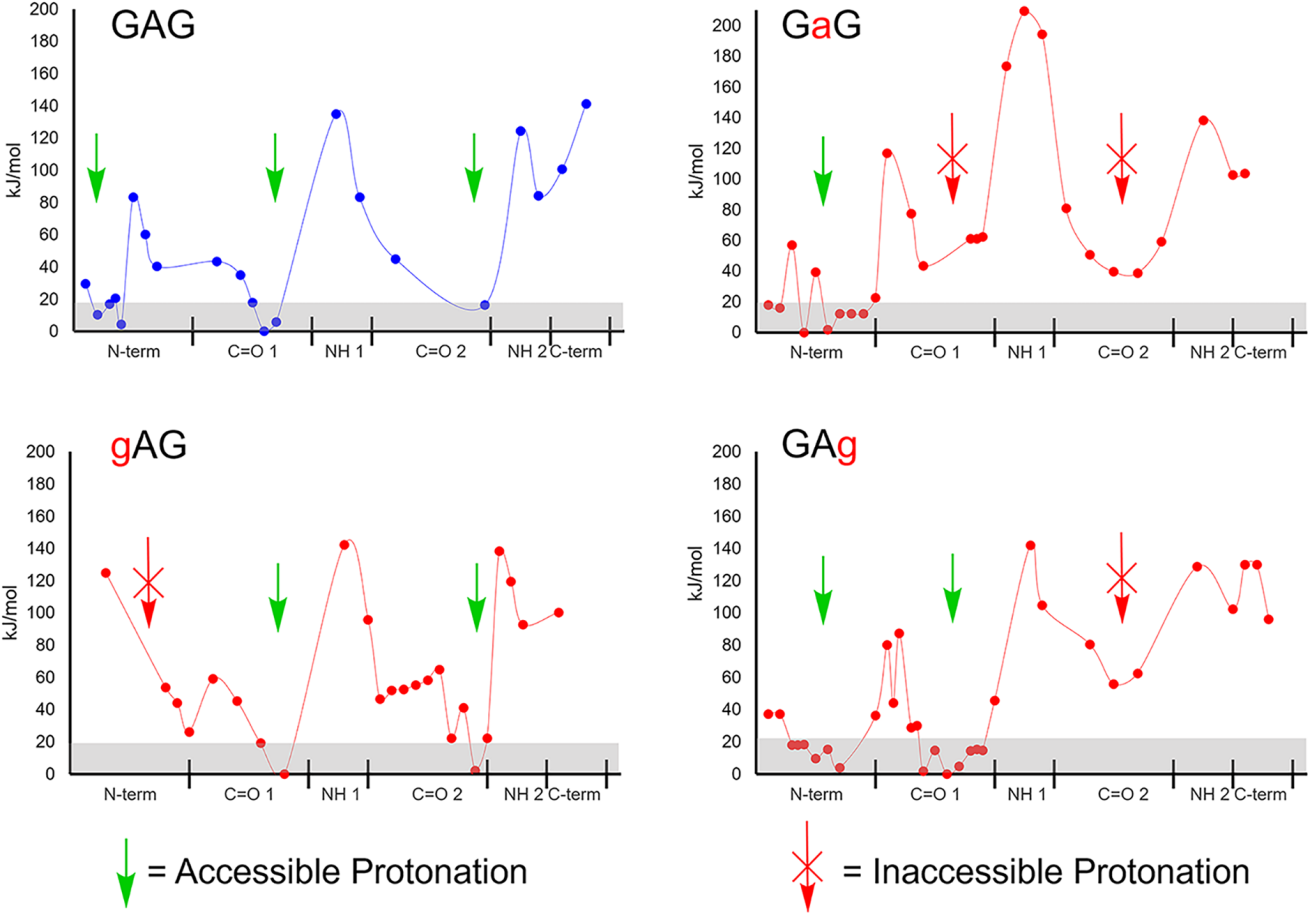
(Depsi)­peptide proton localization geometries (PLGs). The gray band indicates PLGs within 20 kJ/mol of the minimum geometry. Green arrows indicate which backbone sites have sufficient PA to host the mobile proton, and red arrows indicate which do not. The *x*-axis represents the peptide backbone’s heteroatoms, N-term refers to the N-terminus, CO1 to the first residue’s carbonyl, N2 to the amide nitrogen/ester oxygen, etc. The arrangement along the *x*-axis reflects the increasing distance between the backbone heteroatom and the proton.

Differences in the CID threshold energy and MS/MS spectra may be explained in part by PLGs in Figure 3. The gAG depsipeptide (Figure 2b) needed the most energy to drive CID fragmentation. The PLG for gAG in Figure 3 suggests that both amide carbonyls are accessible and the “N” terminus is not. The dominance of the y_2_ fragment ion can be attributed to the higher gas-phase basicity of the Ala over Gly (a.k.a. the y_1_ fragment), consistent with prior insights.^19,21^

GAg (Figure 2d) had the second-highest energy threshold for fragmentation. Compared to the GAG control, it is less favorable for the proton to migrate to the second carbonyl, now an ester, which is the site to initiate b_2_ fragmentation (Figure 3). Therefore, the b_2_ fragmentation of GAg is in competition with y_2_ fragmentation. The b_2_ and y_2_ fragments of GAg track closely until high collision energies.

The GaG depsipeptide (Figure 2c) had a lower fragmentation threshold but was still higher than the GAG control with its fully mobile proton. However, unlike GAG, its b_2_ oxazolone fragment is unfavorable; the central hydroxy acid cannot stabilize a proton. This depsipeptide has an ester linkage between the first and second residue; with no double bond character, it is less stable than amide linkages and cleaves primarily at this site.

An alternative explanation of breakdown curve data in Figure 2 is based on relative stabilities of b_2_ oxazolone ions; b_2_ ions were abundant for both GAG and GAg but not for gAG and GaG. However, an issue with this explanation is that gAG should be able to form a stable b_2_ fragment ion as well as protonation would occur at the central Ala amino acid and not on a hydroxy acid. Yet, for gAG, the b_2_ signal was low (≤10% R.A.); y_2_ was the dominant fragment (Figure 2b).

Calculated geometries for various protonation sites of gAG and GAG are shown in Figure 4. For gAG, protonation at the “N”-terminal hydroxyl is not energetically accessible. Hydrogen bonding stabilizes the proton between the first and second carbonyls; these sites are effectively degenerate (within 2 kJ/mol). Thus, proton mobility appears to be limited for gAG. Additionally, we infer the y_2_ fragment ion of GaG (Figure 2c), which has an “N”-terminal hydroxyl also, could adopt similar conformations as gAG with the proton localized between carbonyls.

**Figure 4:**
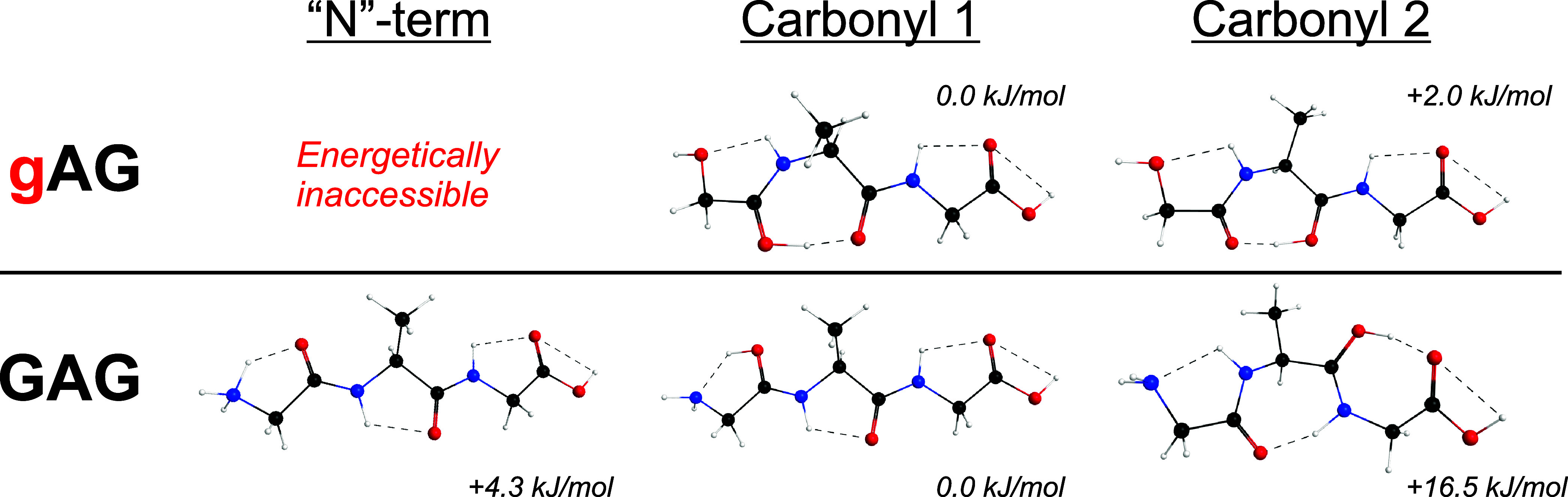
Calculated low-energy geometries of gAG and GAG protonated at different sites. “N” terminal protonation is inaccessible for gAG; its proton is stabilized between the first and second carbonyls. For GAG, protonation between the second and third carbonyls is accessible and appears to be suitable for b_2_ ion formation.

As shown in Figure 3, proton mobility is unhindered for the GAG control. Notably, when GAG is protonated at the second carbonyl, there is a hydrogen bond between the proton and the C-terminus (Figure 4). This orientation of GAG appears to be suited for nucleophilic attack and subsequent b_2_ oxazolone formation. We speculate that this may contribute to observed differences in CID fragmentation between gAG (dominant y_2_ ion) and GAG (dominant b_2_ ion). This argument would be strengthened by more rigorous calculations and/or ion spectroscopy^42,43^ in future work.

In addition to the PA/mobility argument, intramolecular hydrogen-bonding networks suggested by our calculations also provide evidence for qualitative resonance and inductive effects. This can be seen for GaG and GAg (Figure 5), in which internal ester linkages lack the traditional hydrogen bond donor characteristics of amide/peptide linkages. Amide backbone nitrogens can act as hydrogen bond donors, increasing the single bond character of carbonyls and raising their PA. Ester backbone oxygens, however, are poor hydrogen bond donors and cannot distribute electron density in this manner. This causes a divergent ability to participate in hydrogen-bonding networks that increase PA across the backbone. Relatedly, GaG and GAg exhibit antiplanar preferences at internal hydroxy acid residues.

**Figure 5:**
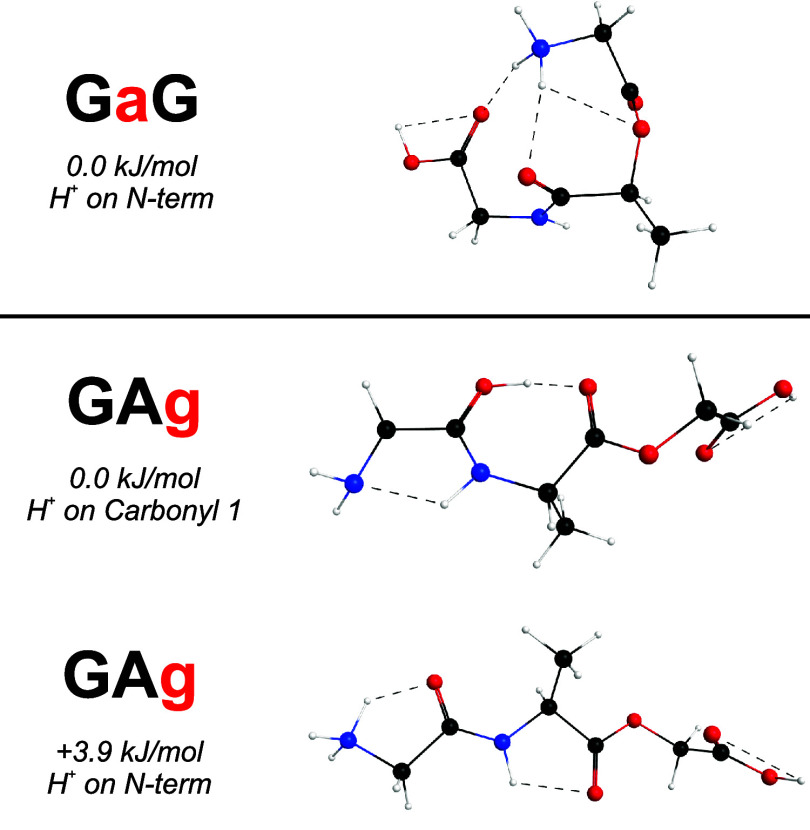
Calculated low-energy geometries of protonated GaG and GAg. Ester linkages have limited participation in hydrogen-bonding networks and reduce the overall planarity.

## Conclusions

Here, CID fragmentation of linear tridepsipeptides was compared to a peptide control. We varied the location of the hydroxy acid residue across the backbone, and the positioning of the hydroxy acid directly influenced protonation, gas-phase stability, and fragmentation. In general, PA decreased at the sequence position of the hydroxy acid residue and at the preceding residue. The depsipeptides studied in this work preferentially generated y-type ions when the hydroxy acid was incorporated at or near the N-terminus and both b- and y-type ions when the hydroxy acid was near the C-terminus. Decreased PA adjacent to hydroxy acid residues can be rationalized with a resonance argument as well as the differing inductive effects that stem from the divergent hydrogen-bonding properties of amides and esters.

From these results, hydroxy acid residues can be understood as charge directors: they do not associate strongly with the charge-carrying proton nor spread this charge with hydrogen bonding across the backbone as amides do. In future work, we seek to incorporate such information into MS/MS sequencing algorithms for various applications.

## Supplementary Material

















## Notes

I. Solution-phase synthesis protocols and schemes; HRMS and NMR characterization of gAG, including all spectra. II. LTQ Velos Pro instrument parameters. III. Full scan and negative mode experiments and spectra. IV. Computational method overview. V. Breakdown threshold data. (PDF)
Jobs 6995-9147, gAG and GAG. (CSV)
Jobs 8976-9147, gAG and GAG. (CSV)
Jobs 9219-9221, gAG. (CSV)
Jobs 9236-9362, GaG. (CSV)
Jobs 9248-9368, GAg. (CSV)
Jobs 9369-9391, GAg. (CSV)
Jobs 9375-9389, GaG. (CSV)

## †

Department of Chemistry, Purdue University, West Lafayette, Indiana 47907, United States

## Abbreviations

MS/MS: tandem mass spectrometry
CID: collision-induced dissociation
LUMO: lowest unoccupied molecular orbital
RRKM theory: Rice–Ramsperger–Kassel–Marcus theory
ESI: electrospray
DFT: density functional theory
PLG: proton localization geometry
LQIT: linear quadrupole ion trap
