# Supplementary material for: Depsipeptide Analogues of Gly-Ala-Gly: Proton Localization and Effects on Collision-Induced Dissociation
Source: J Am Soc Mass Spectrom. 2025 Dec 22;37(1):329–35. doi: 10.1021/jasms.5c00371 (PMC12784392; doi:10.1021/jasms.5c00371)
Supplement: Supplementary file 1 [file js5c00371_si_001.pdf]

Supporting Information for:

Depsipeptide Analogs of Gly-Ala-Gly: Proton Localization and Effects on Collision Induced Dissociation

Brison A. Shira, Elin C. Herndon, Julianna E. DeMauro, Michael W. Giuliano, and Jay G. Forsythe*

Department of Chemistry and Biochemistry, College of Charleston, Charleston, SC USA

*Email: forsythejg@cofc.edu

Table of Contents **Page(s)**

I. Solution-Phase Depsipeptide Synthesis……………………………………S2-S6

Ia. NMR Spectra…………………………………………………..……….S7-S9

II. MS Instrumental Parameters………………………………………………S10

III. Positive Full Scan and Negative Mode Experiments…………………S11-S16

IV. Additional Notes on Computational Methods…………………………S17

V. Breakdown Threshold Data………………………………………………..S18

I. Solution-Phase Depsipeptide Synthesis

Materials and additional notes. Reagents and monomers listed below were used as received without further purification. Boc-L-Alanine was obtained from Novabiochem. 2,2,2-trifluoroacetic acid (TFA; biotech grade), Glycine benzyl ester hydrochloride and N,N-diisopropylethylamine were obtained from Acros. 1-Ethyl-3-(3-dimethylaminopropyl)carbodiimide (EDC) and hydroxybenzotriazole (HOBt) were obtained from Chem-Impex. ACS reagent-grade HPLC-grade CH_3OH was obtained from Fisher. ACS Reagent-Grade dichloromethane (DCM), ACS Reagent-Grade ethyl acetate, NMR solvents, and glycolic acid and 5 wt% Pd/C (wet) were obtained from Millipore-Sigma. NMR tubes were obtained from Bruker. Silica and aluminum-backed TLC plates with F254 visualization were obtained from EMD-Millipore and Silicycle.

NMR Spectra were recorded on a 400 MHz Bruker Avance III Spectrometer equipped with a 5 mm PABBO broadband probe with Z gradients.

Mass spectra were recorded from acetonitrile or methanol solutions by direct infusion in the modes indicated with the spectra below on either a ThermoFisher Velos Pro LTQ system or Orbitrap Elite with ESI sources.

glycolic acid – Ala – Gly (gAG):

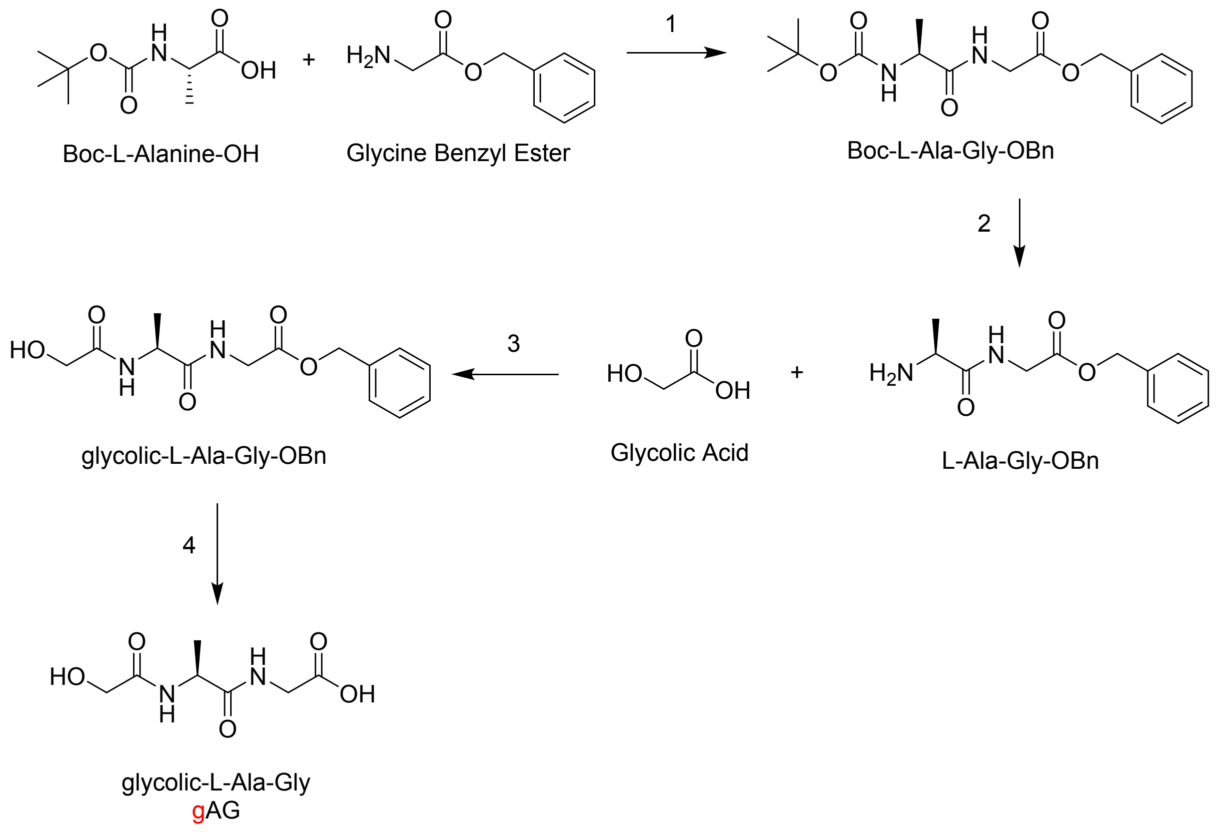

Figure S1. Synthesis of gAG. 1) EDC, HOBt, iPr_2EtN, CH_2Cl_2, 20 h, r.t. (18.3 °C), 60%. 2) TFA (neat), r.t., 45 min. 3) EDC, HOBt, iPr_2EtN, CH_2Cl_2, 20 h, r.t., 28% over 2 steps (isolated after chromatography). 4) H_2, 5% Pd/C (wt., wet), CH_3OH, 3h, r.t. > 95%.

Protocols.

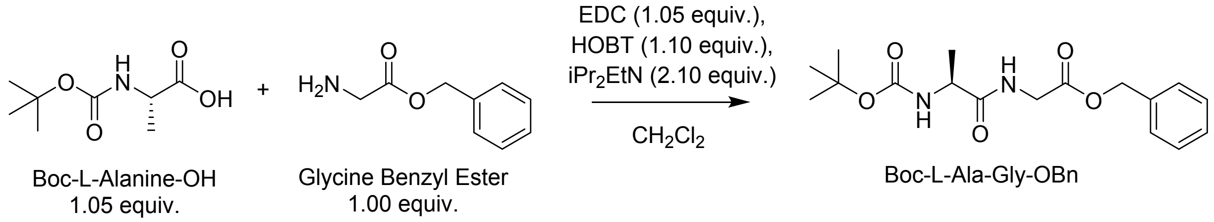

Figure S2. Coupling to produce Boc-L-Ala-Gly-OBn (*Reaction 1*)

Procedure: Boc-L-Alanine (0.492g, 1.05 equiv, 2.60 mmol) was added 50 mL round bottom flask at ambient conditions (room temperature = 18.3 °C). 13 mL DCM was added to afford a 2.0 M solution of amino acid. iPr_2EtN (0.860 mL, 5.21 mmol, 2.1 equiv.), then HOBt (0.369g, 2.73 mmol, 1.1 equiv.), and lastly EDC (0.498g, 2.60 mmol, 1.05 equiv.) were added and the mixture was stirred for 5 minutes following dissolution of reactants. Glycine benzyl ester hydrochloride (0.500g, 2.48 mmol, 1.0 equiv.) was then added and the reaction was stoppered with a septum at stirred for 20 hours at room temperature.

Following the reaction, DCM was removed by rotary evaporation to afford a pale yellow oil. The crude reaction was transferred to a 250 mL separatory funnel by 3 successive 10 mL washes with EtOAc; a small 4 mL portion of DCM was used to solubilize any remaining residue and added to the washes. The crude mixture was then washed successively once each with 20 mL aliquots of 10% (w/v) citric acid (aq), saturated $NaHCO_3$ (aq), and lastly saturated NaCl (aq). The organic layers were dried over $MgSO_4$, solvent was removed by rotary evaporation, and the crude product was dried on high vacuum. ^1H NMR in $CDCl_3$ of an aliquot of this material confirmed successful formation of the dipeptide (*vide infra*, section **Ia** of this supporting information), which was carried forward without further purification. 0.50 g isolated, white amorphous solid, 60% yield. Procedural note: subsequent reproductions of this protocol following this study have yielded up to ~80% with back-extraction of aqueous washes.

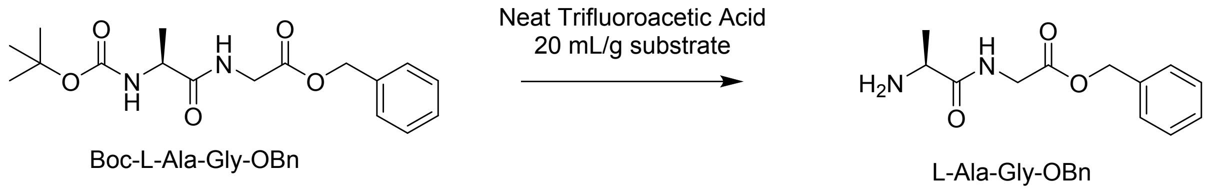

Figure S3. Boc Deprotection. (*Reaction 2*).

*****HAZARD STATEMENT*****

2,2,2-Trifluoroacetic acid (TFA) is a volatile strong organic acid. Extreme care must be taken when handling it. It must only be opened inside a hood as it is an inhalation hazard. Goggles are advised. In the below procedure, care was taken to protect the experimentalist. Two pairs of gloves with liners were worn and the hand was never passed over the lid of the TFA bottle.

Procedure: To a 250 mL round bottom flask containing Boc-L-Ala-Gly-OBn dipeptide (0.50 g, 1.49 mmol, 1.0 equiv.) was added 10 mL neat 2,2,2-triflouroacetic acid (TFA). The flask was loosely

stoppered with a septum at stirred at ambient conditions for 45 minutes. TFA was then blown off via an air stream in the fume hood. The remaining residue was diluted with 10 mL with HPLC-grade water, transferred to a 50 mL conical centrifuge tube, flash-frozen in liquid nitrogen, and solvent was removed via lyophilization. This was insufficient to remove all residual TFA, so the remaining residue was resuspended in DCM (10mL) and concentrated via air stream; this was repeated an additional time, placing the flask in water bath to offset evaporative cooling. The residue was dried over high vacuum for 12 hrs. The crude deprotected dipeptide was carried forward without purification.

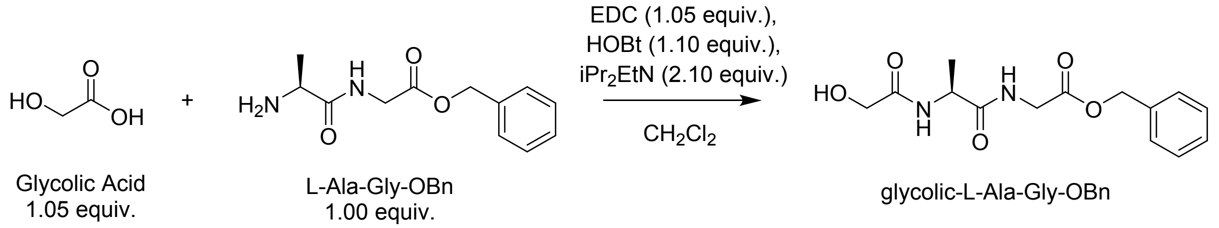

Figure S4: Coupling of glycolic acid to L-Ala-Gly-OBn*TFA. (*Reaction 3*; note that L-Ala-Gly-OBn is present as its TFA salt)

Procedure: **Note:** Special attention was paid to the activation of glycolic acid, which when allowed to proceed for minimal time afforded product as detailed in the absence of hydroxyl protecting groups; extended time at the activation step, even for 5 minutes or more, resulted in oligomerization of glycolic acid before and during the coupling step.

5 mL DCM was added to a round bottom flask containing L-Ala-Gly-OBn*TFA and the mixture was slurried at room temperature. In a separate vial, glycolic acid (0.208g, 2.73 mmol, 1.05 equiv) and HOBt (0.386g, 2.86 mmol, 1.1 equiv.) were dissolved in 5 mL DCM to afford a 0.55 M solution (this concentration difference from the original coupling was mandated by solubility concerns). iPr_2EtN (0.903 mL, 5.46 mmol, 2.1 equiv.) was added in two parts – one to the vial containing glycolic acid (1.05 equiv.) and the other to the flask containing the dipeptide TFA salt, which evolved some TFA vapor and became homogeneous. EDC (0.523g, 2.73 mmol, 1.05 equiv.) was then added to the vial containing glycolic acid. The resulting solution was stirred for *no more than 2 minutes* and then added in one portion to the round bottom flask; the vial was rinsed further into the flask with 10 mL DCM. The flask was stoppered with a septum and then stirred at ambient conditions for 20 hours.

Following reaction, solvent was removed by rotary evaporation, and the remaining residue was transferred to a 250 mL separatory funnel with three 10 mL rinses of EtOAc, followed by a 5 mL rinse with DCM to solubilize any remaining residue. The crude mixture was then washed successively once each with 20 mL aliquots of 10% (w/v) citric acid (aq), saturated $NaHCO_3$ (aq), and lastly saturated NaCl (aq). The organic layers were dried over $MgSO_4$, solvent was removed by rotary evaporation, and the crude product was dried on high vacuum for 12 hours. Crude product identity was confirmed by MALDI-TOF MS on a Voyager DE-STR instrument in reflector mode collected from 200-1000 m/z with 75% grid voltage, a 150 ns delay, laser intensity set to 2500 (arbitrary units), with 100 shots. $[M+H]^+/[M+Na]^+/[M+K]^+$ found: 295.1244/ 317.0954/333.0884 ; $[M+H]^+/[M+Na]^+/[M+K]^+$ calculated: 295.1288/317.1108/333.0847. Notably

absent were peaks associated with protonated or metalated ions at masses 375 or 433 which were associated with multiple additions of glycolic acid.

The crude material was dissolved in ~13 mL 2:1 DCM:EtOAc with a few drops of methanol and loaded onto a silica column (2 cm diameter, ~5 inches height packed silica) packed in 100% EtOAC. The column was eluted first with three column volumes (75 mL total) EtOAc, followed by 70 mL DCM, then 70 mL 5% (v/v) MeOH in DCM. Product ultimately eluted with ~2 column volumes of 10% MeOH in DCM (v/v), observed by TLC (Rf = 0.25, eluted with neat EtOAc). 110 mg isolated, 28% yield over 2 steps; material carried forward after 1H and 13C NMR confirmation of product, final characterization and purity estimate performed after final debenzylation step below. We note this procedure lacks protection of the glycolic acid hydroxyl, which saves additional purification and deprotection steps in the synthetic route.

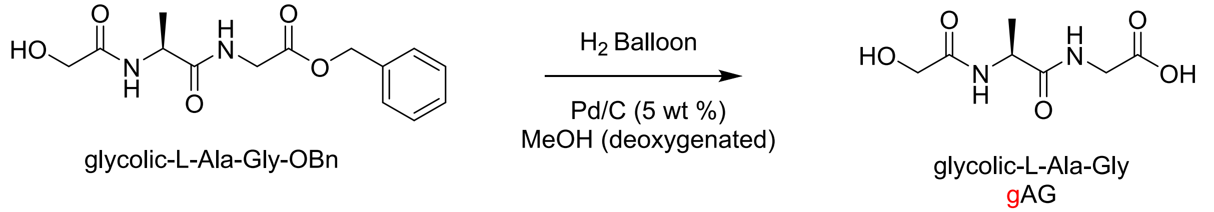

Figure S5. Hydrogenation to yield final gAG product. (*Reaction 4*)

Procedure: 30 mL HPLC-grade MeOH was purged of oxygen with nitrogen gas bubbling for 39 mintues in a 100 mL roundbottom flask. Protected depsipeptide gAG-OBn (30 mg, 0.10 mmol, 1.0 equiv) was added to the MeOH, followed by a catalytic amount (~2 spatula tips) of 5 wt% Pd/C (wet). The flask was sealed under nitrogen with a septum and parafilm. H2 was added to the reaction via 2 balloons, and the reaction was then stirred for 3 hours at room temperature (18.3 °C). The reaction mixture was then filtered through celite topped with a small amount of silica into a roundbottom flask. MeOH was then removed via rotary evaporation and the final product was dried on high vacuum. 20 mg isolated, > 95% yield, >95% purity (est. from ^{1}H NMR).

Characterization of Final Product:

HRMS of gAG (Orbitrap Elite; positive ion mode). Spectrum was collected via direct infusion from acetonitrile solution of gAG final product.

Ion	*Theo. m/z*	*Exp. m/z*	*ppm error*
$[M+H]^+$	205.0819	205.0813	-2.9
$[M+Na]^+$ (base peak)	227.0638	227.0632	-2.6

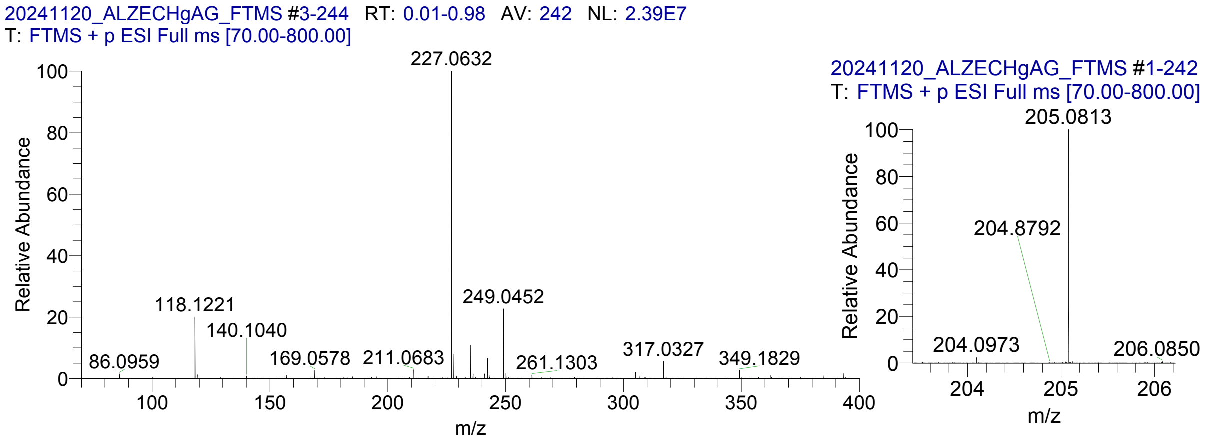

Figure S6. HRMS of final gAG product. $[M+Na]^+$ is base peak. (*Inset* = $[M+H]^+$ ion).

Chemical Shift Assignments:

^{1}H NMR assignments were taken from a standard 1D ^{1}H spectrum in DMSO-*d6.* The limited solubility of gAG in this solvent (and complete insolubility in many other standard NMR solvents) precluded direct measurement of its ^{13}C NMR spectrum. However, all resonances were assigned from 2D ^{1}H-^{13}C HMQC and HMBC experiments, all collected using standard Bruker pulse sequences. Data were processed in Mestrenova, and spectra for crude reaction monitoring and final product analysis are included in the following section. All spectra were referenced internally to solvent residual peaks.

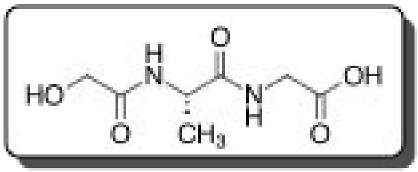

	**Resonance**	(ppm)					
**Residue**	*HN*	*Hα*	*Hβ*	*OH*	*C*	*Cα*	*Cβ*
glycolic acid(1)	n/a	3.82	n/a	5.80	171.37	60.96	n/a
Alanine(2)	7.87	4.32	1.23	n/a	171.08	47.26	18.24
Glycine(3)	7.42	3.27	n/a	n/a	170.51	43.71	n/a

Notes: C = carbonyl carbon; OH for carboxylate not observed due to exchange

Ia. NMR Spectra and Assignments from gAG synthesis

^{1}H 1D, $CDCl_3$, 400 MHz *reaction 1 crude product*

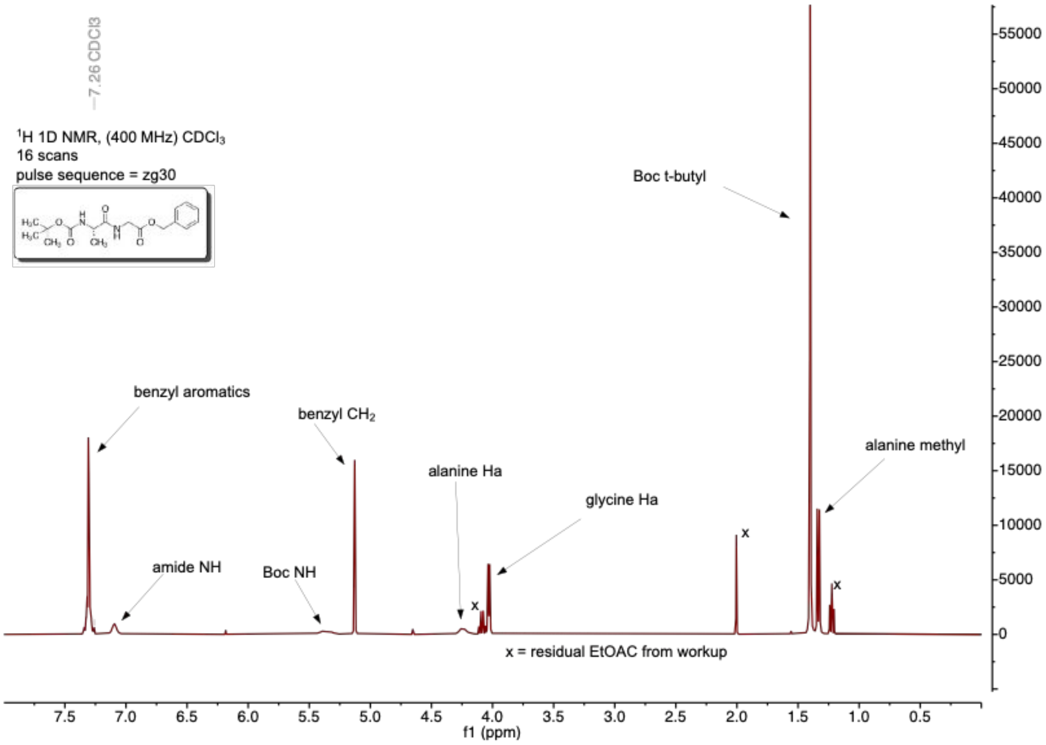

^{1}H 1D, $CDCl_3$, 400 MHz, *reaction 3 product after flash chromatography*

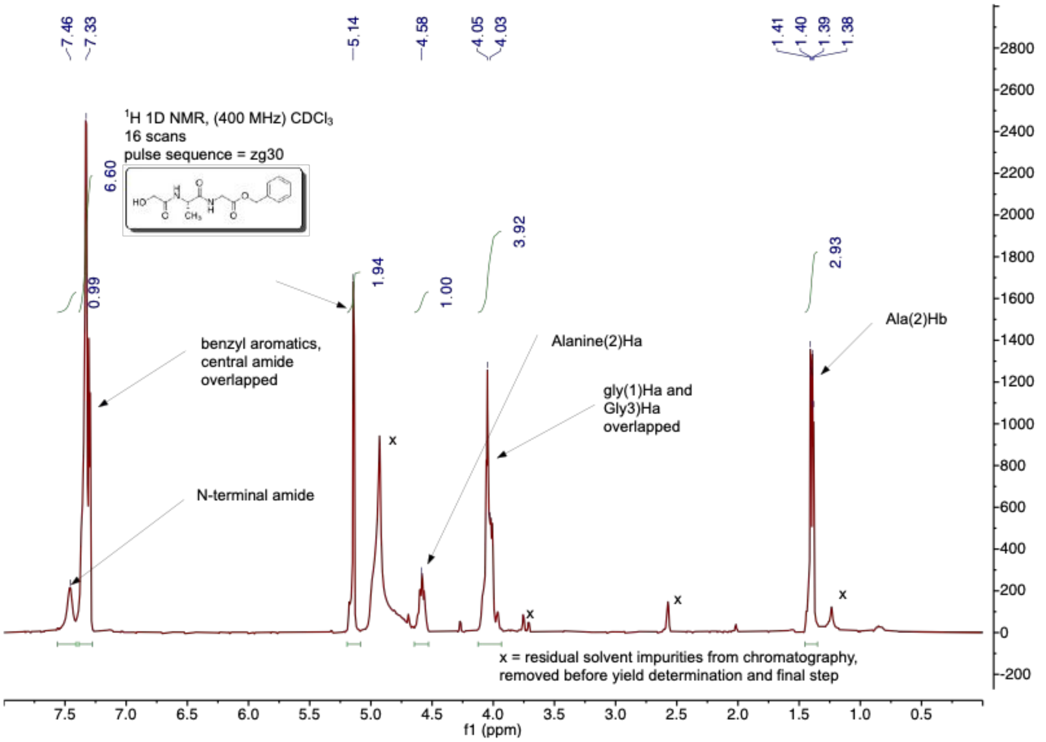

^{13}C 1D, $CDCl_3$, 100 MHz, *reaction 3 product after flash chromatography*

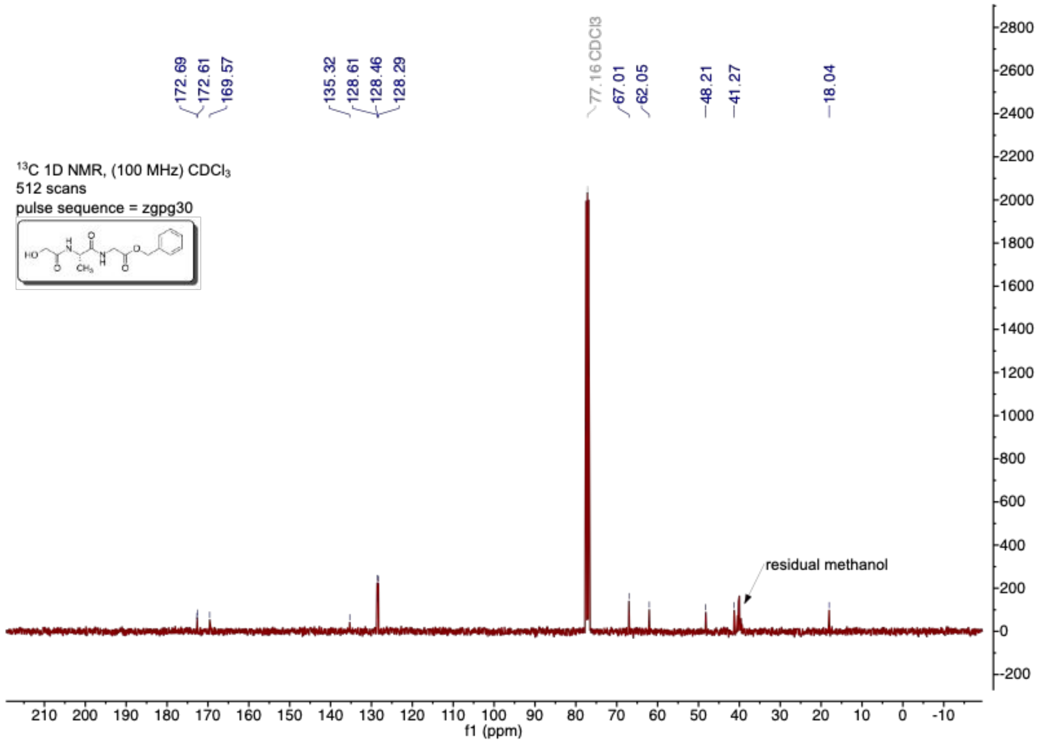

^{1}H 1D, dmso-*d6*, 400 MHz, *final gAG product*

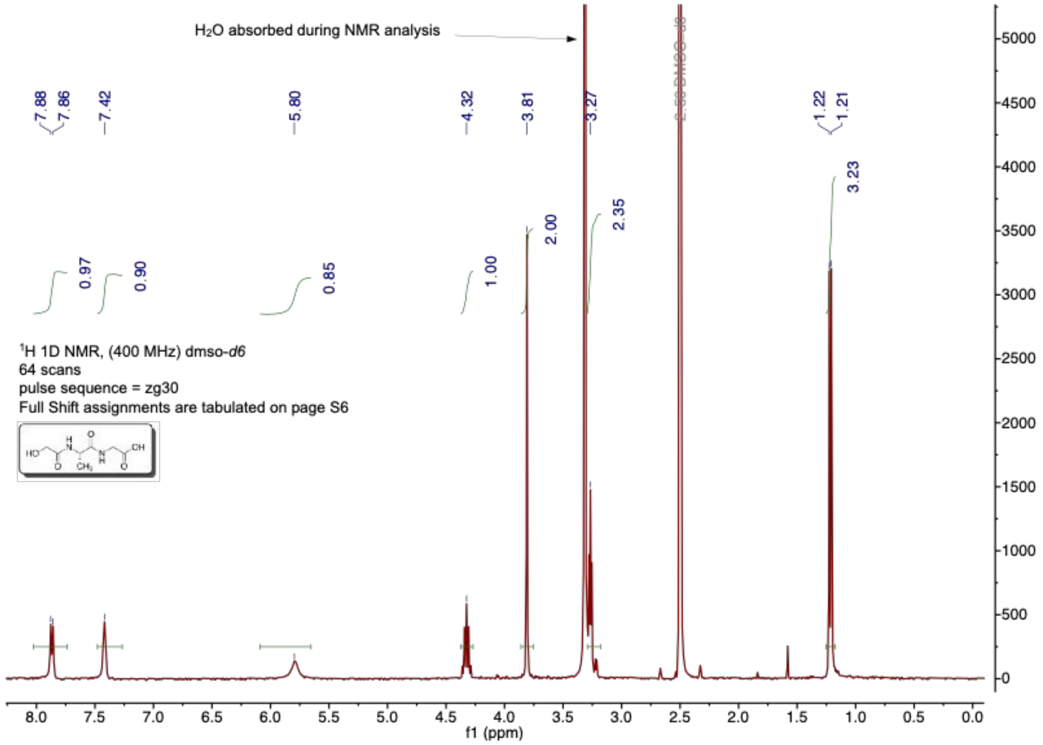

^{1}H-^{13}C 2D phase-sensitive HMQC, dmso-*d6*, (400 MHz, 100 MHz) *final gAG product*

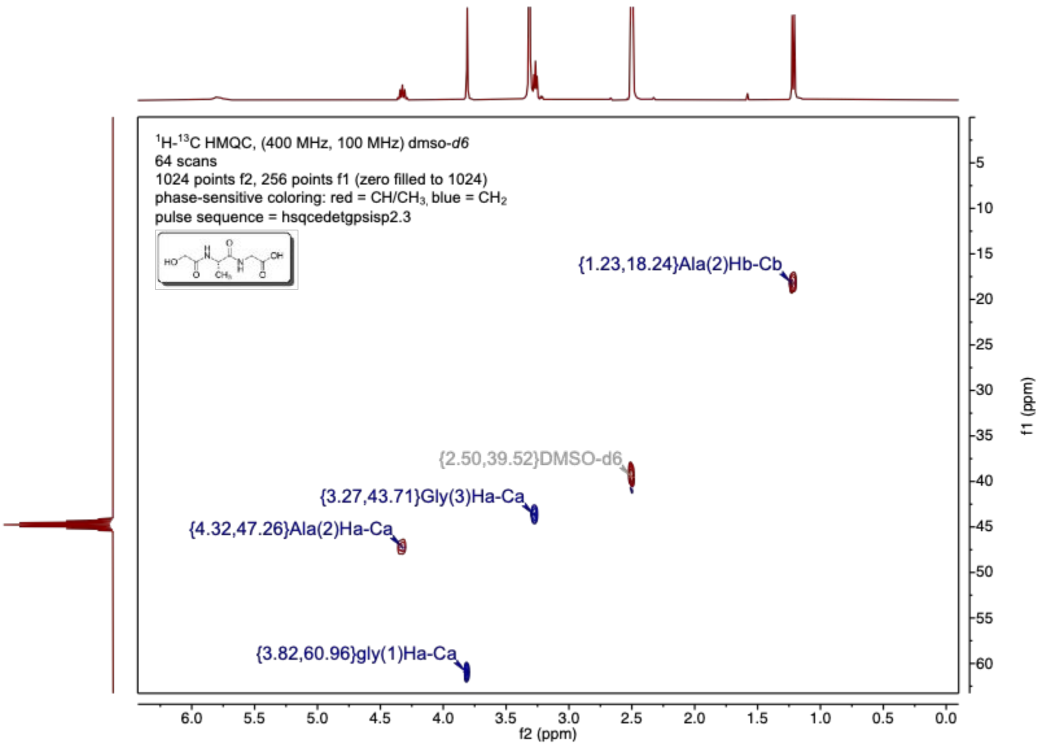

^{1}H-^{13}C 2D HMBC, edited for $^{2}J_{CH}$ dmso-*d6*, (400 MHz, 100 MHz) *final gAG product*

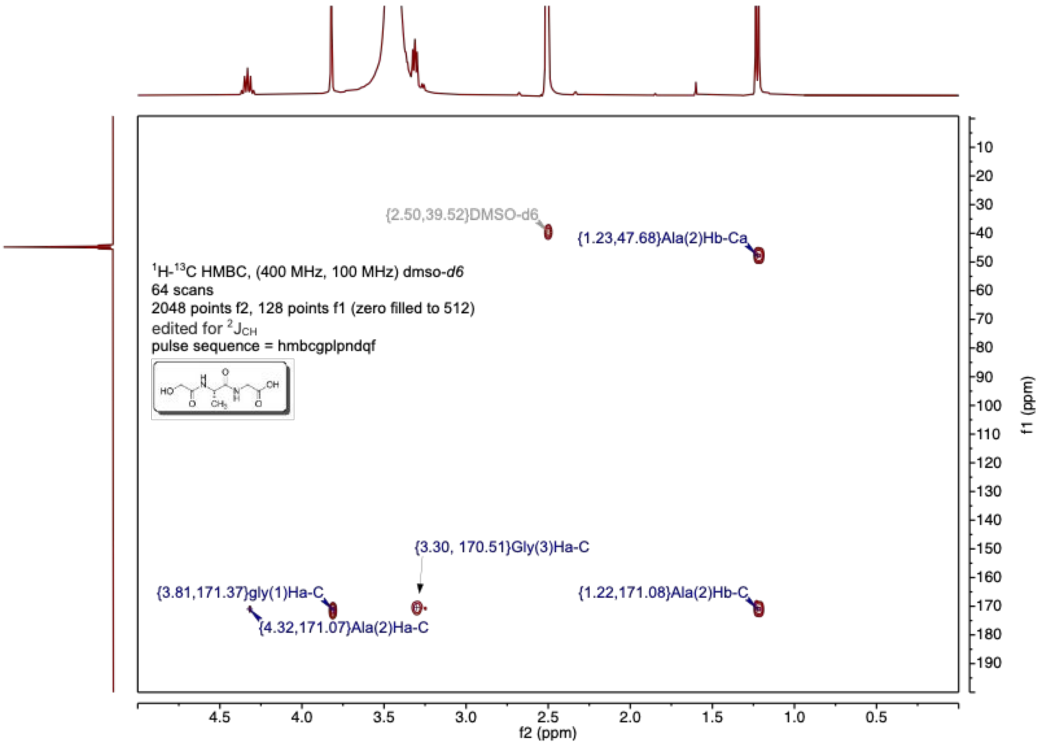

II. MS Instrumental Parameters

Positive ion mode:

Samples were infused into the ESI source at 4.0 µL/min flow rate for all spectra. The spray voltage was 4.5 kV. The LTQ had the following ionization source settings: temperature 333 K, sheath gas flow 20 arb, auxiliary gas flow 5 arb, sweep gas flow 5 arb, capillary temperature 523 K, S-lens RF level 45.0%. This instrument uses a He bath gas.

For MS/MS experiments, we accumulated spectra at a given collision energy for 60 seconds and tabulated the observed relative abundance of the fragments over that time. The activation q was set to 0.250 (which is the default on this instrument).

Negative ion mode:

The same settings were used as above, except the spray voltage was set to (-) 2.0 kV; rather than the (+) 4.5 kV used in positive mode. The results of these experiments are presented in **S3** below.

III. Positive Full Scan and Negative Mode Experiments (LTQ Velos Pro)

All mass spectra below were recorded as described in **II**.

Gly-Ala-Gly (GAG control):

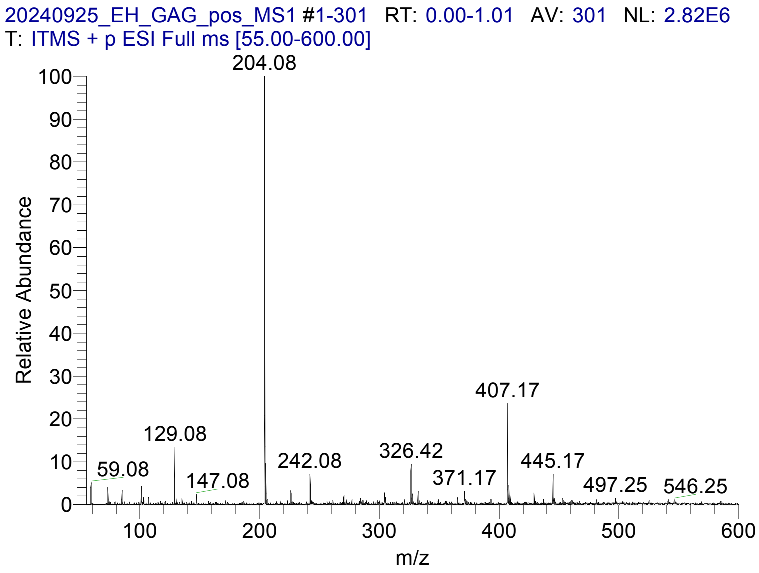

Figure S7. GAG positive ion mode, full scan.

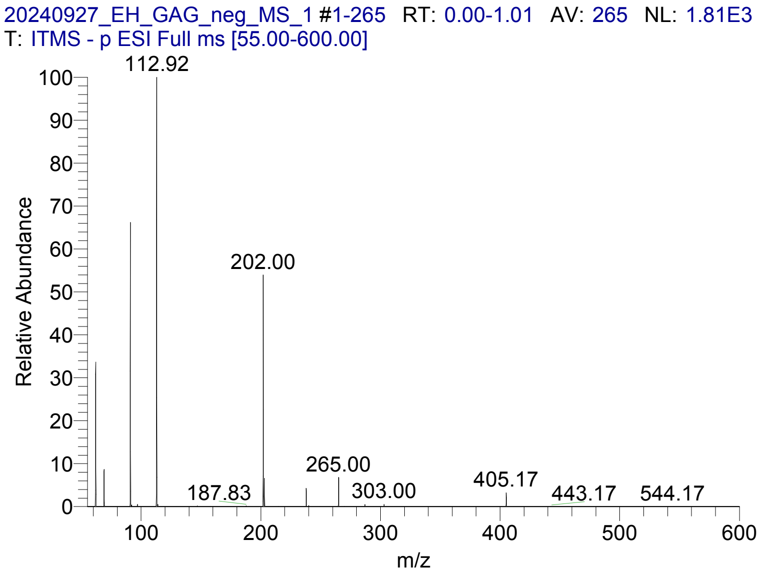

Figure S8. GAG negative ion mode, full scan.

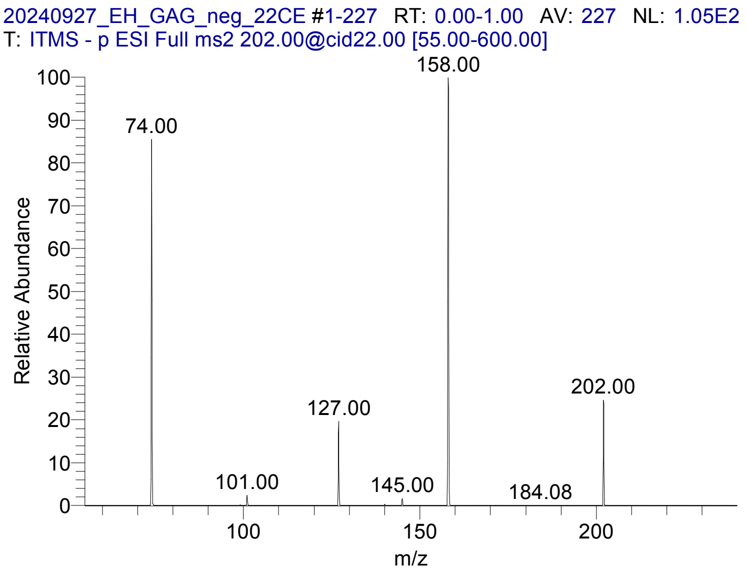

Figure S9. GAG negative ion mode, MS/MS (CE = 22 V).

glycolic acid – Ala – Gly (gAG):

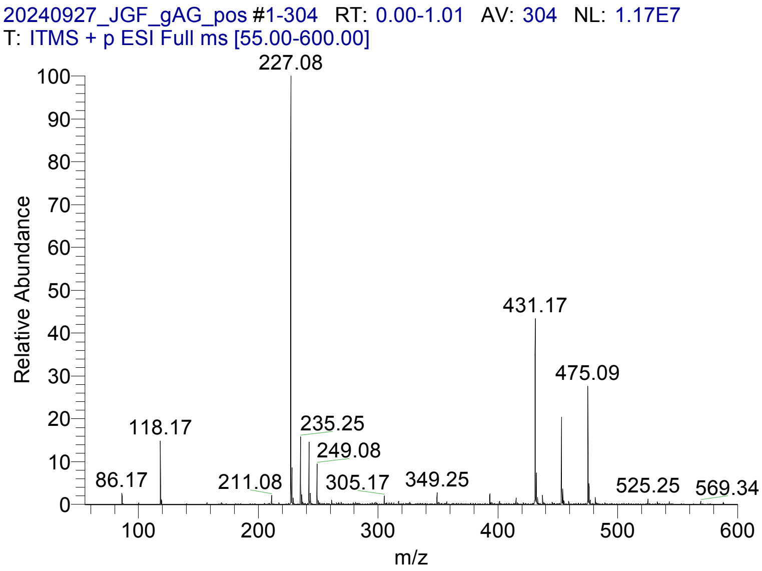

Figure S10. gAG positive ion mode, full scan.

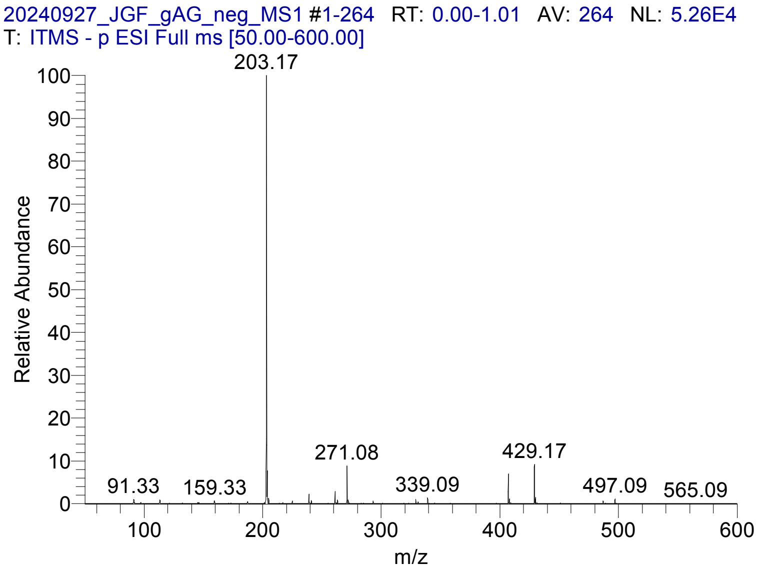

Figure S11. gAG negative ion mode, full scan.

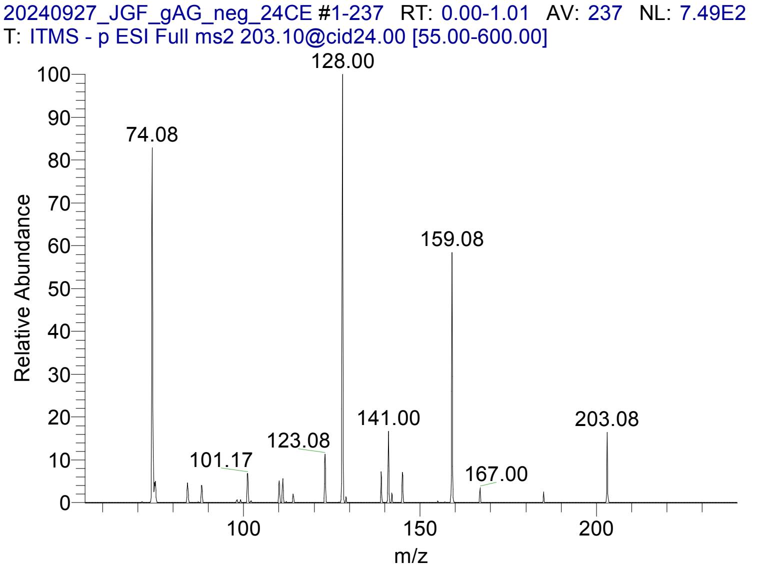

Figure S12. gAG negative ion mode, MS/MS (CE = 24 V).

Gly – lactic acid – Gly (GaG):

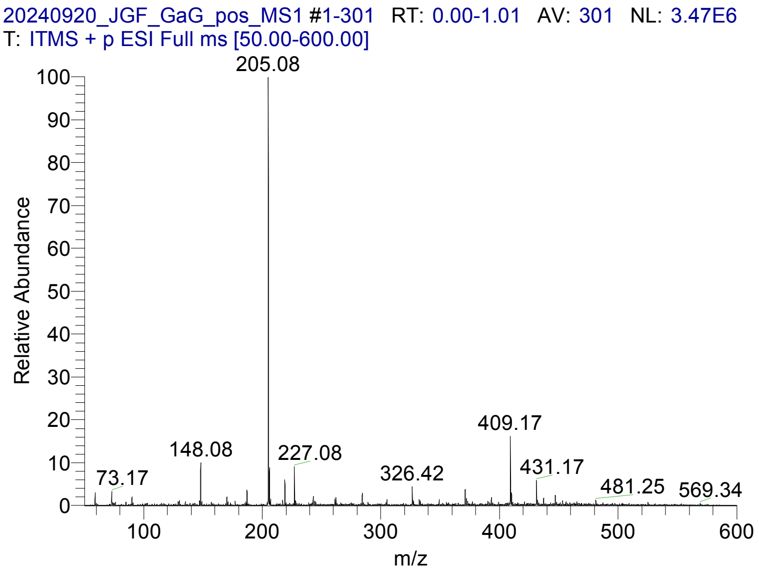

Figure S13. GaG positive ion mode, full scan.

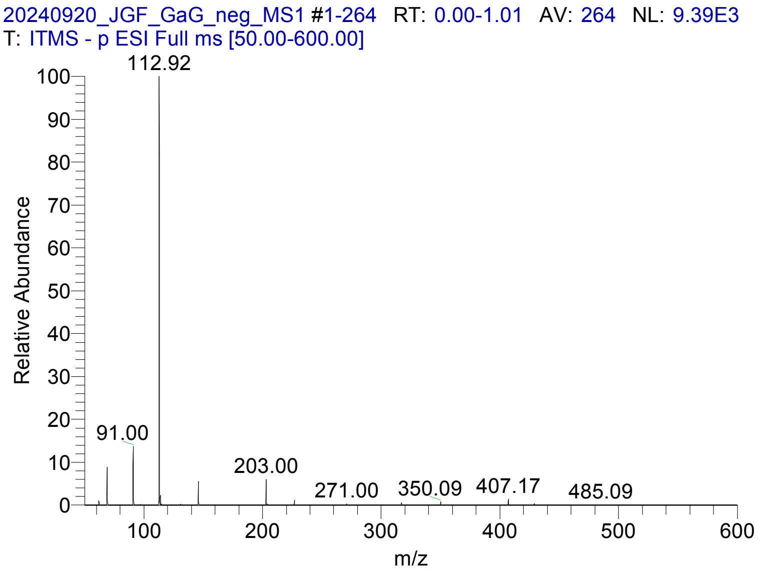

Figure S14. GaG negative ion mode, full scan.

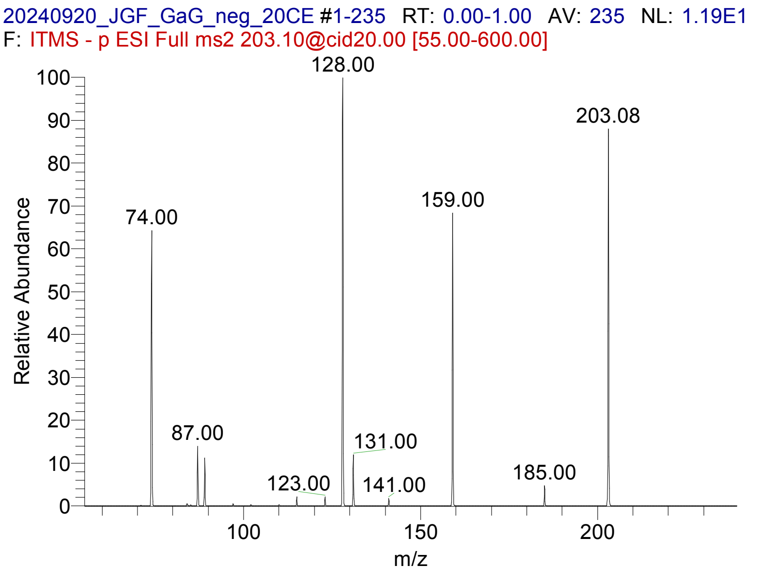

Figure S15. GaG negative ion mode, MS/MS (CE = 20 V).

<u>Gly – Ala – glycolic acid (GAg):</u>

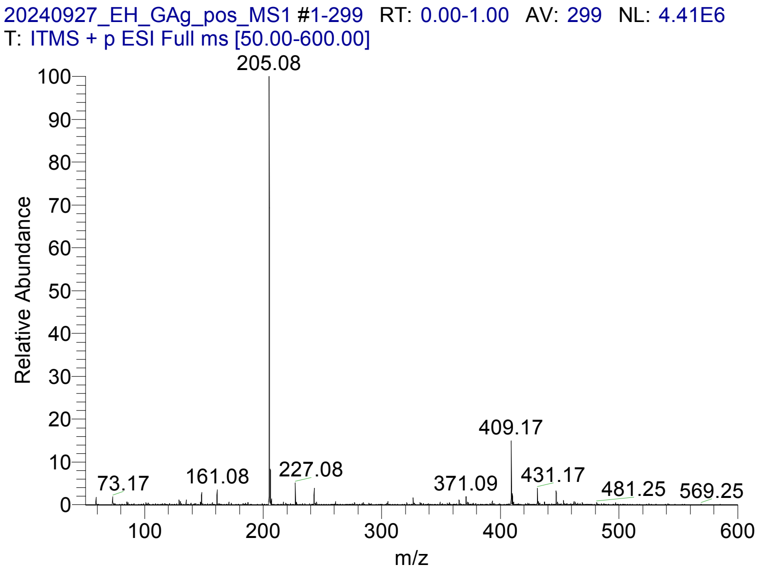

Figure S16. GAg positive ion mode, full scan.

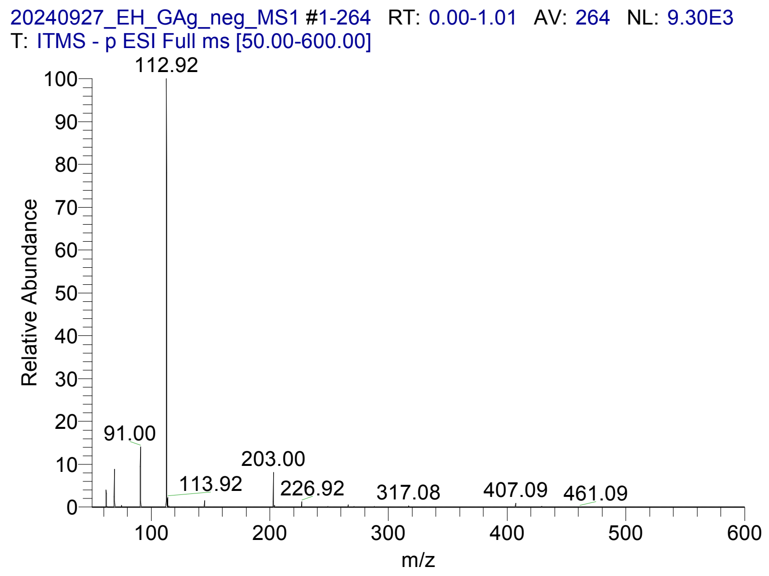

Figure S17. GAg negative ion mode, full scan.

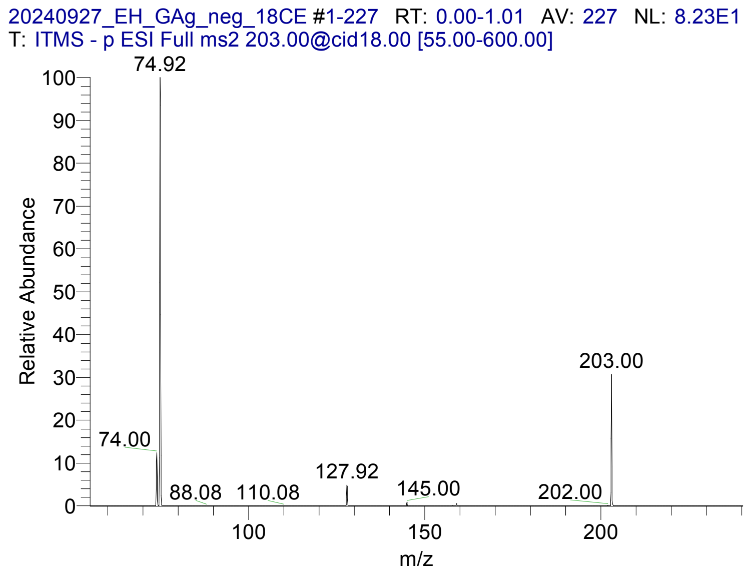

Figure S18. GAg negative ion mode, MS/MS (CE = 18 V).

IV. Additional Notes on Computational Methods

The methods presented here were used to calculate the accessible protonations along the peptide backbone as described in the main text.

We first used a semi-empirical geometrization (PM6) to begin to envision each of the polar groups associated with a proton. We then minimized these structures and variants using B3LYP/6-311+G(2d,p) and followed these geometry minimizations with higher level energy calculations (see below). We tabulated these results to depict a potential energy surface of the proton associated with each of the different backbone heteroatoms.

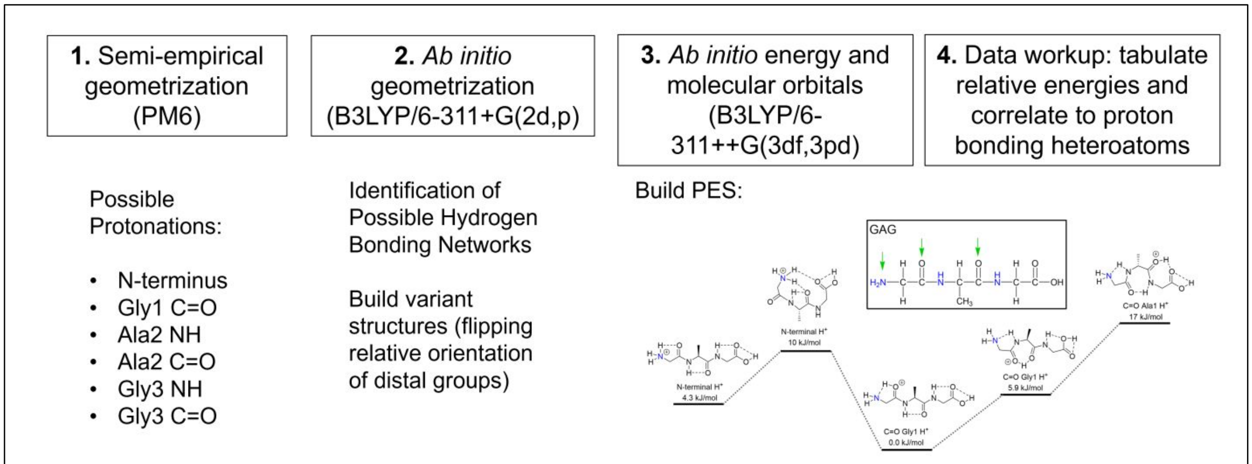

Figure S19. A flow chart depicting the stages of calculations. Ideally, variant geometries will be generated for each possible protonation and optimized. Low energy geometries from other species modeled should also be substituted to ascertain the stability difference, or whether a minimum was missed (i.e., if a gAG low energy conformer is identified, its N-terminus can be substituted and the same geometry can be optimized for GAG, expanding the potential energy surface, or PES, characterization).

V. Breakdown Threshold Data

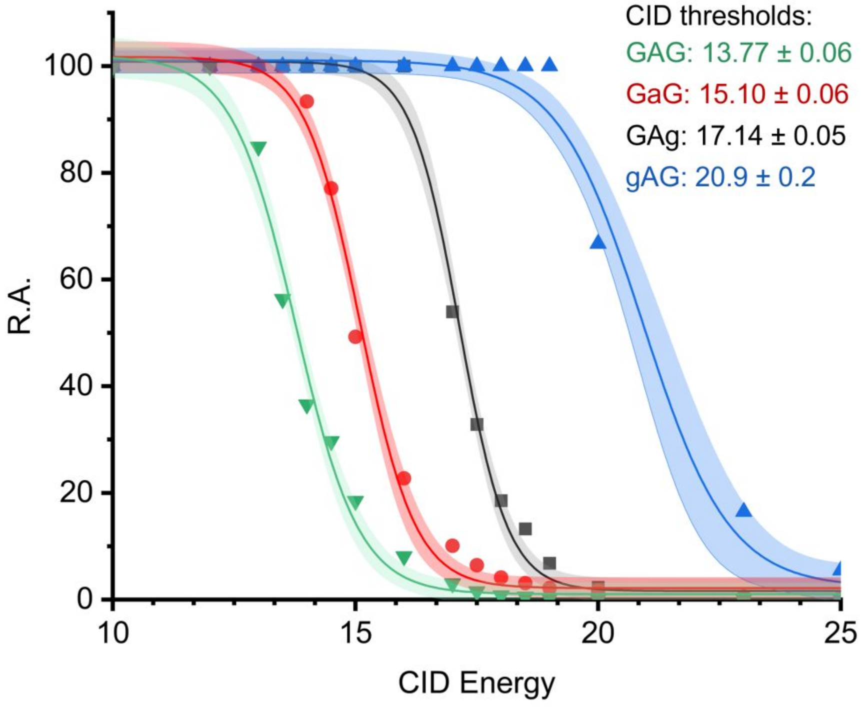

Figure S20. Breakdown curve data (main text **Figure 2**) for the precursor $[M+H]^+$ ions of each analyte (*m/z* 204 and 205) are presented in direct comparison. These data are here fitted to a Boltzmann function (solid curves). The R-square value for each fit was ≥ 0.992. Though the fit was performed for the entire data range, only CID Energy from 10-25 are shown. The shaded regions represent a 95% confidence interval about the fitted curve. From the fitted curves the x_0 value was determined (the CID energy at which the parent ion has 50% RA). This value is the relative CID threshold; a measure of how much CID energy is required to fragment the analyte. These values are tabulated in the top right. Curve fitting allows for error analysis (*i.e.,* 95% confidence intervals and error on x_0), showing that the use of breakdown curves in this study is a high-confidence method to probe the CID fragmentation processes accessible to each isomer.
